# The socio-economic determinants of infant mortality in Nepal: analysis of Nepal Demographic Health Survey, 2011

**DOI:** 10.1186/s12887-015-0468-7

**Published:** 2015-10-12

**Authors:** Khim Bahadur Khadka, Leslie Sue Lieberman, Vincentas Giedraitis, Laxmi Bhatta, Ganesh Pandey

**Affiliations:** Save the Children, Kathmandu, Nepal; Department of Anthropology, University of Central Florida, Orlando, FL 32816-0955 USA; Faculty of Economics, Vilnius University, Vilnius, Lithuania; Tribhuvan University, Birendra Multiple Campus, Bharatpur, Nepal

**Keywords:** Socioeconomic factors, Proximate determinants, Infant mortality, Nepal

## Abstract

**Background:**

Infant mortality reflects not only the health of infants but societal well-being as a whole. This study explores distal socioeconomic and related proximate determinants of infant mortality and provides evidence for designing targeted interventions.

**Methods:**

Survival information on 5391 live born infants (2006–2010) was examined from the nationally representative Nepal Demographic Health Survey 2011. Bivariate logistic regression and multivariate hierarchical logistic regression approaches were performed to analyze the distal-socioeconomic and related proximate determinants of infant mortality.

**Results:**

Socio-economic distal determinants are important predictors for infant mortality. For example, in reference to infants of the richest class, the adjusted odds ratio of infant mortality was 1.66 (95 % CI: 1.00–2.74) in middle class and 1.87 (95 % CI: 1.14–3.08) in poorer class, respectively. Similarly, the populations of the Mountain ecological region had a higher odds ratio (aOR =1.39, 95 % CI: 0.90–2.16) of experiencing infant mortality compared with the populations of the Terai plain region. Likewise, the population of Far-western development region had a higher adjusted odds ratio (aOR =1.62, 95 % CI: 1.02–2.57) of experiencing infant mortality than the Western development region. Moreover, the association of proximate determinants with infant mortality was statistically significant. For example, in reference to size at birth, adjusted odds ratio of infant dying was higher for infants whose birth size, as reported by mothers, was very small (aOR = 3.41, 95 % CI: 2.16–5.38) than whose birth size was average. Similarly, fourth or higher birth rank infants with a short preceding birth interval (less than or equal to 2 years) were at greater risk of dying (aOR =1.74, 95 % CI: 1.16–2.62) compared to the second or third rank infants with longer birth intervals. A short birth interval of the second or the third rank infants also increased the odds of infant death (aOR = 2.03, 95 % CI: 1.23–3.35).

**Conclusions:**

Socioeconomic distal and proximate determinants are associated with infant mortality in Nepal. Infant mortality was higher in the poor and middle classes than the wealthier classes. Population of Mountain ecological region and Far western development region had high risk of infant mortality. Similarly, infant dying was higher for infants whose birth size, as reported by mothers, was very small and who has higher birth rank and short preceding birth interval. This study uniquely addresses both broader socioeconomic distal and proximate determinants side by side at the individual, household and community levels. For this, both comprehensive, long-term, equity-based public health interventions and immediate infant care programs are recommended.

## Background

Infant mortality rate is defined as the risk of a live-born child to die before its first birthday. Infant mortality rates reflect economic and social conditions for the health of mothers and newborns, as well as the effectiveness of health systems [1]. The causes of infant mortality are strongly correlated to structural factors, like economic development, general living conditions, social wellbeing, and the quality of the environment, that affect the health of entire populations [2]. In industrial world, a dominant factor in the decline in infant mortality has been social and economic progress [3]. Therefore, in a scenario where the infant mortality rate is declining, the social, economic or demographic determinants assume important roles. In Nepal demographic variables, previous birth interval and survival of the preceding child predominated as determinants of infant mortality, particularly in rural areas of Nepal [4].

Millennium Development Goal 4 aims for a two-thirds reduction in infant mortality by the year 2015 [5]. In Nepal it is declined by 42 % over the last 15 years and is on track to achieve Millennium Development Goal 4 [6]. Infant mortality was 46 per 1000 live births during the period 2006–2010 [7]. However, not all segments of the society equally benefited from the progress that was made and many impoverished people in Nepal are struggling with poor health care [8]. Regional and district inequity observed in the budget allocations have contributed to inequitable health outcomes [9]. Similarly, there is a huge rural to urban disparity reflected in the physician to population ratio of 1:850 in capital city Kathmandu and 1:30,000 outside of the capital [10]. According to the Mosley-Chen framework, socio-economic factors at the community, household or individual levels operate through five proximate determinants and are the pathways through which socio-economic processes affect infant health [11]. Therefore, this study aims to explore the role of distal socio-economic and related proximate determinants of infant mortality at different levels in Nepal.

## Methods

### Data sources

This study analyzed the secondary data from the nationally representative Nepal Demographic Health Survey (NDHS), 2011 accessed from the Measure Evaluation Demography Health Survey 2011 Nepal [12]. The Enumeration Area (EA) was defined as a ward in the rural areas and a sub-ward in the urban areas. In Nepal, Village Development Committees (VDCs) are considered as rural and Municipalities as urban area. There are nine wards in a VDC, and the number of wards ranges from nine to 35 in municipalities. Stratification was achieved by separating each of the 13 domains into urban and rural areas. The number of wards and sub-wards in each of the 13 domains were not allocated proportional to their populations due to the need to provide estimates with acceptable levels of statistical precision for each domain; and for the urban and rural domains of the country as a whole. The vast majority of the population in Nepal resides in the rural areas. In order to provide for national urban estimates, urban areas of the country were over sampled. In each stratum, samples were selected independently through a two-stage selection process. In the first stage, EAs were selected using a probability proportional-to-size strategy. In order to achieve the target sample size in each domain, the ratio of urban EAs over rural EAs in each domain was roughly 1 to 2, resulting in 95 urban and 194 rural EAs (289 EAs). Due to the non-proportional allocation of the sample to the different domains and to over sampling of the urban area in each domain, sampling weights are considered to ensure the actual representativeness of the sample at the national level as well as the domain levels.

### Conceptual framework

**Fig. 1:**
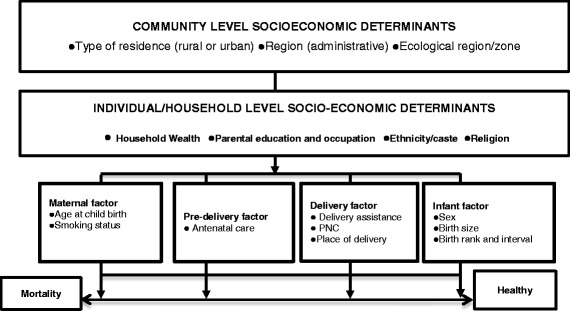
Conceptual framework of determinants influencing infant mortality

The Mosley and Chen conceptual framework for the study of child survival in developing countries (Fig. 1) [11] was adapted based on the available information in the 2006–2010 NDHS datasets. Table 1 gives the selection and classification of variables used in this study in view of the conceptual framework.

**Table 1:** Operational definition, categorization and dummy coding of the variables

Variables/ Determinants	Definition and categorization
COMMUNITY LEVEL	
Ecological region	Ecological zone (1 = Mountain, 2 = Hill and 3 = Terai (plain area/Lowlands))
Region (administrative)	Developmental regions (1 = Far western, 2 = Mid western, 3 = Eastern, 4 = Central and 5 = Western)
Residence	Type of residence (0 = Rural, 1 = Urban)
HOUSEHOLD LEVEL	
Household wealth index	Composite index of household amenities (1 = Poorest, 2 = Poorer, 3 = Middle, 4 = Richer and 5 = Richest)
Maternal ethnicity/caste	Maternal ethnicity/caste (1 = Dalit, 2 = Janajati, 3 = Others, 4 = Brahmin, Chettri and Newar)
Maternal religion	Maternal religion (1 = Hindu, 2 = Buddhist, Muslim, Christian and Kirat)
Maternal education	Maternal formal years of schooling (0 = No formal school education, 1 = Primary education ie up to class five, 2 = Secondary and higher education ie above class five)
Father’s education	Paternal formal years of schooling (0 = No formal school education, 1 = Primary education ie up to class five, 2 = Secondary and higher education ie above class five)
Mother’s occupation	Mother’s occupational status (0 = Not working, 1 = Official (professional, technical, managerial and clerical), 2 = Sales and services, 3 = Skilled manual, 4 = Unskilled manual and 5 = Agriculture)
Father’s occupation	Father’s occupational status (1 = Official (professional, technical, managerial and clerical), 2 = Sales and services, 3 = Skilled manual, 4 = Unskilled manual and 5 = Agriculture)
PROXIMATE LEVEL	
Sex of infant	Sex of infant (0 = Male and 1 = Female)
Birth size	Subjective assessment of the respondent on the birth size (1 = Very large, 2 = Larger than average; 3 = Smaller than average, 4 = Very small and 5 = Average)
Birth rank and birth interval	Birth rank and birth interval of baby (1 = 1st birth rank, 2 = 2nd or 3rd birth rank and birth interval ≤ 2 years; 3 = ≥ 4^th^ birth rank and birth interval >2 years, 4 = ≥ 4th birth rank, birth interval ≤2 years; 5 = 2nd or 3rd birth rank and birth interval >2 years)
Age of mother at child birth	Maternal age at child birth (1 = <20 years, 2 = 20 to 35 years of age)
Antenatal care visit	Antenatal service received by the mother ((0 = No and 1 = Yes, any visit)
Use of tobacco	Use tobacco by mother (0 = No, smokes nothing and 1 = Yes but did not cover the frequency and duration of smoking)
Place of delivery	Place of delivery (0 = Home and 1 = Health facility)
Delivery assistance	Birth attendance during delivery (0 = By Traditional Birth Attendant/other and 1 = By Skill Birth Attendant or health professional)
Post Natal Check up (PNC) visits	Postnatal check up visits (0 = No, 1 = Within 24 h and 2 = 1 day to 45 days)
OUTCOME LEVEL	
Death of infant	Death of infant (0 = No and 1 = Yes)

#### Key explanatory variables

The outcome was infant death, which is the death of a live born infant in the first year of life. In this analysis, it was re-coded as a binary variable. The explanatory variables included community level distal socioeconomic, ​the household and individual level socioeconomic determinants and proximate determinants, covering maternal, infant, pre-natal, delivery, and post-natal factors in line with conceptual framework of study.

#### Community level socioeconomic determinants

People living in municipalities including towns and the capital city were considered as urban people and people living in villages or rural areas were considered as rural people. Development regions covered five administrative regions while ecological regions covered Mountain, Hill and Terai ecological zones.

#### The household and individual level socioeconomic determinants

In this study, the main socioeconomic determinant is household wealth quintile (index). It is a method developed by the ORC Macro to measure the socioeconomic level for a household in a ranked order. It uses principal-component analysis based on respondents’ household assets, amenities, and services [13]. In the 2011 NDHS, this variable covered information on material possessions (e.g., television, bicycle car), as well as dwelling characteristics such as source of water, sanitation facilities and type of material used in flooring [13]. The individual’s rank is based on their household score and divided into quintiles where the first quintile is the poorest 20 % of the households and fifth quintile is the wealthiest 20 % of the households [14]. Similarly, categorical or ordinal variables; no formal school education, primary education, secondary education and higher education are used for mother’s and father’s education level. The other variables consist of sex of the child, ethnicity and religion of mother. Ethnic/caste groups with similar characteristics are categorized. Religion of the mother is categorized into two categories: Hindu and others (Buddhist, Christian, Kirat, and Muslim). Age of mother, while giving childbirth is categorized into two groups (less than 20 and 20 year to 35 years of age).

#### The intermediate or proximate determinants

The proximate determinants include birth size, birth order and previous birth interval. Size at birth (very small, small, average size, large or very large) was obtained by asking mothers. Birth rank was categorized into three groups: first, 2–3 birth rank and 4^+^-birth rank. The preceding birth interval was grouped into two groups: less than 2-year and two or more years. These two variables are combined into one variable with categories [15]. First rank, 2–4 birth rank with 2-years or more of preceding spacing, 2–3 birth rank with less than 2-years of preceding spacing, 4^th^ or more birth order with 2-year or more of preceding spacing and 4^+^ birth rank with less than 2-years of preceding spacing.

### Data analysis

A synthetic cohort life table approach was used to calculate infant mortality rate. Data were weighted by sampling probabilities to represent the structure of Nepali population using weighting factors provided with the NDHS [16]. Due to incomplete exposure for death, births in the month of interview were excluded from the analysis.

Frequency tabulations were used to describe the data, followed by the bivariate analysis using Chi-square tests and contingency table analyses to examine the association of all potential determinants on infant mortality without adjusting for other covariates. Prior to multivariate hierarchical logistic regression analysis, multi-collinearity between the variables was assessed and variables with multi-collinearity were not considered for the analysis. For example, parental education level and occupation were highly correlated with wealth index, so these variables were not considered in the analysis though they were significant. In addition, only those variables that were significant in the bivariate analysis were further analyzed using multivariate hierarchical logistic regression. A p-value less than 0.05 was considered as significant and odds ratios at 95 per cent confidence intervals were determined.

Based on a conceptual framework describing the hierarchical relationships between different groups of variables, multivariate hierarchical logistic regression was used to assess the association of distal socioeconomic and proximate determinants on infant mortality after controlling other variables. In this approach the associations of more distal variables can be examined without improper adjustment by proximate or intermediate variables that may be mediators of the effects of more distal variables [16]. At the initial stage, community level variables were entered in the model and only those that were significantly associated with infant mortality were retained in the first model. In the second stage, the socioeconomic level variables were added to the first model, and only the significant variables were retained to assess the association of the socioeconomic level variables in the presence of community level variables. In the last stage, the proximate determinants were entered into the second model, and the associations of the significant proximate determinants were assessed in the presence of both socioeconomic and community level variables. Negelkerke pseudo R^2^ was used to assess goodness-of-fit of logistic models. The Statistical Package for Social Science (SPSS 16.0 for Windows) software was used to analyze the data.

The Nepal DHS 2011 was approved by the ethics review board of the ICF Macro International and the Ministry of Health and Population. All respondents were verbally informed before consenting to their participation. This research study ensured that their participation was voluntary and independent when answering and interacting with the interviewers. The researcher also maintained confidentiality of the information, which was received after permission from Measure DHS.

## Results

This research included 5391 live-births, occurring within the 5 years preceding the survey. The characteristics of explanatory variables are given in Table 2. The most infants (53 %) were from the Terai ecological region while only 8 % of the infants were from the Mountain ecological region. Around 32 % of infants were from the Central development region whereas 11 % of infants were from the Far-western development region. A majority (91 %) of infants were from rural areas. About 26 % of the infants were from the poorest household, compared to about 14 % of infants from the richest household. The majority (66 %) of infants were average in size at birth. About 6 % of infants were born with a > = 4 birth rank & a birth interval of = <2 years.

**Table 2:** Infant mortality rate (per 1000 live births), 5 year periods preceding the survey and unadjusted Odds Ratio by explanatory variables (*n* = 5391, weighted)

Determinants	*n*, weighted	Percent	IMR [95 % CI]	Bivariate logistic regression		
				uOR	95 % CI	
Ecological Region						
Mountain	428	7.9	65 [49–80]	1.45*	1.04	2.03
Hill	2131	39.5	45 [35–55]	1.12	0.84	1.50
Terai	2833	52.5	44 [34–55]	1	-	-
Development Region						
Far western	605	11.2	66 [49–83]	1.74*	1.11	2.71
Mid western	793	14.7	42 [30–53]	1.18	0.75	1.87
Eastern	1269	23.5	46 [32–61]	1.17	0.74	1.85
Central	1717	31.8	45 [31–60]	1.11	0.70	1.78
Western	1007	18.7	40 [25–55]	1	-	-
Type of place of residence						
Rural	4888	90.7	47 [40–55]	1.30	0.92	1.82
Urban	503	9.3	40 [26–54]	1	-	-
Wealth Index						
Poorest	1390	25.8	49 [38–60]	2.53***	1.51	4.21
Poorer	1182	21.9	50 [35–66]	2.21**	1.28	3.80
Middle	1133	21.0	55 [37–73]	2.11**	1.21	3.67
Richer	937	17.4	41 [25–57]	1.90*	1.07	3.38
Richest	748	13.9	29 [14–45]	1	-	-
Ethnicity						
Dalit	958	19.8	52 [34–71]	1.05	0.73	1.50
Janajati	1751	36.2	49 [36–60]	1.05	0.77	1.43
Other (Muslim and Terai other caste)	410	8.5	51 [23–82]	1.21	0.69	2.11
Brahmin Chhetri and Newar	1713	35.5	38 [30–46]	1	-	-
Religion						
Hindu	4466	82.8	47 [40–55]	1.18	0.81	1.72
Others	926	17.2	42 [25–58]	1	-	-
Highest educational level of mother						
No education	2550	47.3	55 [44–66]	1.55*	1.15	2.10
Primary	1079	20.0	44 [30–58]	1.35	0.93	1.97
Secondary and higher	1763	32.7	36 [25–46]	1	-	-
Highest education level of father						
No education	1243	23.2	65 [48–83]	1.71**	1.26	2.32
Primary	1312	24.5	51 [36–65]	1.32	0.97	1.79
Secondary and higher	2801	52.3	36 [28–45]	1	-	-
Mother occupation						
Not working	1552	28.8	44 [31–59]	0.74	0.54	1.02
High official work	147	2.7	20 [4–40]	0.38	0.12	1.20
Sales and service	315	5.8	38 [11–66]	0.59	0.32	1.09
Skilled manual	107	2.0	65 [14–80]	1.64	0.78	3.43
Unskilled manual	103	1.9	19 [2–49]	0.62	0.23	1.70
Agriculture	3164	58.7	50 [41–58]	1	-	-
Father occupation						
Official type	873	16.6	49 [31–69]	0.80	0.53	1.22
Sales, services	1237	23.5	33 [23–44]	0.73	0.50	1.06
Skilled manual	951	18.1	52 [33–72]	0.85	0.56	1.28
Unskilled manual	833	15.9	58 [40–76]	1.17	0.81	1.70
Agriculture	1361	25.9	45 [32–57]	1	-	-
Sex of the child						
Male	2780	51.6	46 [37–56]	1.06	0.83	1.37
Female	2611	48.4	46 [37–56]	1	-	-
Size of child at birth						
Larger than average	958	17.8	38 [24–51]	0.89	0.62	1.28
Smaller than average	661	12.3	42 [25–61]	0.91	0.61	1.36
Very small	196	3.6	138 [81–190]	3.22***	2.11	4.92
Average	3569	66.3	44 [36–53]	1	-	-
Birth rank and birth interval						
First birth rank	1833	34.0	47 [36–59]	1.44*	1.04	1.99
2–3 birth rank & = <2 years of birth interval	567	10.5	72 [45–100]	2.07**	1.37	3.12
> = 4 birth rank & > 2 years of birth interval	895	16.6	31 [18–45]	0.99	0.65	1.52
> = 4 birth rank & = <2 years of birth interval	296	5.5	81 [43–120]	2.37**	1.46	3.85
2–3 birth rank and >2 years birth interval	1794	33.3	39 [28–51]	1	-	-
Age of mother at child birth						
<20 years	2941	54.6	53 [43–63]	1.27	0.98	1.65
20–35 years	2450	45.4	39 [30–48]	1	-	-
Antenatal visit						
No	609	30.9	49 [29–70]	0.93	0.60	1.44
Yes	1363	69.1	53 [37–68]	1	-	-
Use of tobacco						
No	687	12.7	49 [33–65]	1.32	0.95	1.83
Yes	4704	87.3	46 [38–53]	1	-	-
Place of delivery						
Home	3402	64.1	49 [40–58]	1.31	0.99	1.73
Health Facility	1905	35.9	38 [28–49]	1	-	-
Delivery assistance						
By TBA/Others	3542	65.7	49 [41–58]	1.26	0.95	1.65
By SBA/Health professional	1849	34.3	41 [30–52]	1	-	-
Postnatal check of visits						
No	2221	53.8	36 [26–44]	3.23**	1.62	6.42
Within 24 h	1094	26.5	22 [10–33]	1.99	0.93	4.27
1 day to 45 days	816	19.7	11 [03–19]	1	-	-
National total	**53915391**		**46**			

*** = *p* < 0.001; ** = *p* < 0.01and * = *p* < 0.05, *uOR* unadjusted Odds Ratio, *CI* Confidence Interval

Infant mortality rate was found to be 46 per 1000 live births between 2006 and 2010. IMR was 44 deaths per 1000 live births in the Terai ecological region, compared to 65 deaths per 1000 live births in the Mountain region. It was highest in the Far-western development region (66 deaths per 1000 live births) and lowest in the Western development region (40 deaths per 1000 live births). Similarly, IMR was higher (47 deaths per 1000 live births) in rural areas than in urban areas (40 deaths per 1000 live births). The IMR was 55 in the middle class households compared to 29 in the wealthiest households. IMR was 138, as compared with 38 in larger than average birth size. However, rest of the categories had no significant differences in infant mortality with average size at birth. IMR was 72 in 2–3 birth rank & = <2 years of birth interval and 81 in > =4 birth rank & = <2 years of birth interval category.

Table 2 also shows the crude odds ratios of the explanatory variables associated with infant mortality. This study found a wide variation in the odds of infant death by ecological zone and administrative developmental regions. The higher unadjusted odds of infant death was found in mountain ecological region (uOR = 1.45, 95 % CI: 1.04–2.03) with reference to Terai ecological region. Similarly, there was higher unadjusted odds of infant death in Far western development region (uOR = 1.74, 95 % CI: 1.11–2.71) with reference to Western development region. Likewise, in reference to infants of the Richest class, the unadjusted odds ratio of infants dying of Richer (uOR = 1.90, 95 % CI: 1.07–3.38), Middle (uOR = 2.11, 95 % CI: 1.21–3.67), Poorer (uOR = 2.21, 95 % CI: 1.28–3.80) and Poorest class (uOR = 2.53, 95 % CI: 1.51–4.21) was increased, respectively. In reference to average sized babies at birth, unadjusted odds ratio of infant dying was higher for infants whose birth size according to the mother was very small (uOR = 3.22, 95 % CI: 2.11–4.92). Similarly, the unadjusted odds ratio of infant mortality for fourth or higher birth rank infants with a short preceding birth interval (less than or equal to 2 years) was high (uOR = 2.37, 95 % CI: 1.46–3.85) compared to the second or third rank infants with longer birth intervals. A short birth interval of the second or the third rank infants also showed an increased odd of infant deaths (uOR = 2.07, 95 % CI: 1.37–3.12).

Compared to infants born to mothers who have no formal education or are illiterate, the unadjusted odds of dying was higher for infants whose mothers have secondary and higher levels of formal education (uOR =1.55, 95 % CI: 1.15–2.10). Similarly, compared to infants born to fathers who have no formal education or are illiterate, the unadjusted odds of dying was higher for these infants compared to those whose fathers have secondary and higher levels of formal education (uOR =1.71, 95 % CI: 1.26–2.32). However, parental education level variables were not entered into the model simultaneously as they were found to be highly correlated to the wealth index (Table 2).

In the first model of multivariate hierarchical logistic regression, community level socio-economic determinants had associations with infant mortality. The Mountain ecological region had a higher adjusted odds ratio (aOR =1.39, 95 % CI: 0.90–2.16) of experiencing infant mortality compared with the Terai plain/low land region. Similarly, the Far-western development region had a higher adjusted odds ratio (aOR =1.62, 95 % CI: 1.02–2.57) of experiencing infant mortality than with reference to the Western development region.

The second model presents the results after adding the wealth index as a socioeconomic determinant of infant mortality. Even after inclusion of this variable in model 2, the association of community level determinants with infant mortality was retained for example, the adjusted odds ratio of infant death was 1.33 in mountain ecological region with reference to the Terai ecological region. Similarly, the adjusted odds ratio of infant death was 1.58 in the Far western development region with reference to the Western development region. Furthermore, in reference to infants of the Richest class, the adjusted odds ratio of infant dying was 1.66 (95 % CI: 1.00–2.74) in Middle class and 1.87 (95 % CI: 1.14–3.08) in Poorer class respectively.

The third model presents the results after adding all the proximate determinants. The reduction of the significance level of socioeconomic determinants (wealth index) after inclusion of the proximate determinants (i.e. size of the baby at birth and birth rank and birth interval) indicates that distal determinants are important predictors for infant mortality. For example, in reference to infants of the Richest class, the adjusted odds ratio of infant dying increased to 1.72 (95 % CI: 1.03–2.87) in Middle class and 1.95 (95 % CI: 1.18–3.24) in Poorer class, respectively. Similarly, the association of proximate determinants with infant mortality was statistically significant. In reference to average sized babies, adjusted odds ratio of infant dying was higher for infants whose birth size according to the mother was very small (aOR = 3.41, 95 % CI: 2.16–5.38). Similarly, the adjusted odds ratio of infant mortality for fourth or higher birth rank infants with a short preceding birth interval (less than or equal to 2 years) was higher (aOR =1.74, 95 % CI: 1.16–2.62) compared to the second or third rank infants with longer birth intervals. A short birth interval of the second or the third rank infants also showed increased odds of infant deaths (aOR = 2.03, 95 % CI: 1.23–3.35) (Table 3).

**Table 3:** Multivariate hierarchical logistic regression results by determinants for infant mortality in the 5 years preceding the survey-adjusted Odds Ratio

Determinants	Model I			Model II			Model III		
	aOR	95 % CI		aOR	95 % CI		aOR	95 % CI	
Ecological Region									
Mountain	1.39	0.90	2.16	1.33	0.84	2.10	1.37	0.86	2.17
Hill	1.03	0.78	1.36	1.03	0.76	1.40	1.05	0.77	1.44
Terai	1	-	-	1	-	-	1	-	-
Development Region									
Far western	1.62*	1.02	2.57	1.58	0.99	2.53	1.49	0.92	2.40
Mid western	1.00	0.62	1.61	0.97	0.59	1.58	0.88	0.53	1.45
Eastern	1.16	0.77	1.77	1.16	0.76	1.77	1.07	0.70	1.64
Central	1.14	0.77	1.70	1.14	0.76	1.71	1.10	0.73	1.65
Western	1	-	-	1	-	-	1	-	-
Wealth Index									
Poorest				1.55	0.92	2.61	1.56	0.91	2.68
Poorer				1.66**	1.00	2.74	1.72**	1.03	2.87
Middle				1.87**	1.14	3.08	1.95**	1.18	3.24
Richer				1.40	0.82	2.38	1.43	0.83	2.46
Richest				1	-	-	1	-	-
Size of child at birth									
Larger than average							0.87	0.60	1.26
Smaller than average							0.90	0.59	1.36
Very small							3.41***	2.16	5.38
Average							1	-	-
Birth rank and birth interval									
First birth rank							1.28	0.92	1.78
2–3 birth rank & = <2 yrs of birth interval							1.74**	1.16	2.62
> = 4 birth rank & > 2 yrs of birth interval							0.68	0.43	1.08
> = 4 birth rank & = <2 yrs of birth interval							2.03**	1.23	3.35
2–3 birth rank and >2 yrs birth interval							1	-	-
−2 Log likelihood	2013			2006			1950		
Nagelkerke R Square	0.005			0.009			0.038		

*** = *p* < 0.001; ** = *p* < 0.01and * = *p* < 0.05, *aOR* adjusted Odds Ratio, *CI* Confidence Interval

Negelkerke R^2^ value has increased from model I to model III however its value is low. It suggests that the strength of the association between dependent and independent variable has increased in the successive models.

## Discussion

Analyses of the 2006–2010 Nepal Demographic Health Survey data have revealed consistent relationships between socioeconomic determinants such as wealth of the household and infant mortality. Specifically, middle and poorer classes were vulnerable for infant mortality. Other literature also shows that poor infants are more likely to be exposed to health risks than their better-off peers, and they have less resistance to disease because of under-nutrition and other hazards typical in poor communities. These inequities are compounded by reduced access to preventive and curative interventions. Rich people frequently benefit even from public subsidies for health more than poor people [17]. In addition, there are important practices that are shaped by socioeconomic and environmental influences associated with infant mortality. For example, maternal stress is correlated with premature delivery and lower birth weights both of which are leading causes of infant mortality [18]. Similarly, religious and culturally prescribed and proscribed rules have been practiced in certain ethnic groups may decrease heterozygosity, increase inbreeding and the risk for genetic anomalies leading to increased risk for infant mortality [19]. A recent study in Gaja Strip found that consanguineous marriage was the strongest intermediate factor of infant mortality [20]. Infant mortality decreases with increasing parental education level [21] and better paying occupations which increases household income resulting in higher levels of family consumption and healthier environments. The impact of father’s formal education surpassed mother’s formal education in explaining infant mortality [22]. Similarly, Nepal Fertility and Family Planning Survey (1986) showed significant effects of access to toilets in lowering infant mortality. Nepali’s are experiencing increased access to resources like remittances, toilets and literacy campaigns may reduce the relative impact of these variables on infant mortality. For example, the share of households with access to drinking water (piped to the house) increased from 14 to 22 % from 2004 to 2010 [23]. A reduction in the odds of infant death was observed as the sanitation condition of household increased. Access to a flush toilet was a proxy for household socioeconomic status, which suggests that education and household resources were complementary in lowering the infant mortality [24]. However, in this study, parental education, occupation and environmental-related variables were not included in the analysis model as they were highly correlated with and part of the wealth index.

The majority of infants in this study were from rural areas and infant mortality rates were found to be higher in the rural areas than in urban areas. However, bivariate analysis showed that infant mortality was not statistically significant between rural and urban residence in this period. This indicates the effects of public health program interventions have focused in rural areas. Nepal Health Sector Program II (2010–2015) has targeted to reduce infant mortality at 34 per 1000 live births [25].

Similarly, differences in terms of regional variation were not statistically significant. Likewise, the findings of this analysis showed that sex of the infants did not influence the odds of dying but the literature shows females have lower odds of mortality than males during the first month of life [26–29]. There is evidence from some parts of South Asia that male children receive preferential treatment in terms of better nutrition or health care from their parents [30]. Hence, finding no sex differences in mortality may be due to the large proportion of infants’ deaths occurring in the first week of birth, which is the time when the effects of gender differences in mortality are not pronounced. In the other hand, the finding is supported by the increasing trend of the gender parity index in Nepal. That is a positive indication of focused response in addressing gender disparity issues.

All above-mentioned socioeconomic determinants operated through a common set of significant proximate determinants of infant deaths. These determinants were size of babies at birth and birth rank and birth interval. Smaller infant size at birth was found to be one of the strongest determinants of infant mortality. This finding is supported by other literature as well. Low birth weight was a strong predictor of neonatal mortality [31]. Food-availability also influences child survival by influencing the nutrients available to infants [11]. Tackling the immediate causes of low birth weight should be linked to community-based efforts to deal with the underlying causes of low birth weight, rooted in household and community practices. Hence, further reductions in infant mortality require that maternal nutrition and health issues be addressed. Whilst such programs should be carefully monitored and evaluated, it must be recognized that child survival is reflected throughout the life cycle of women [32]. Furthermore, smoking is also a risk factor that has direct implication in low birth weight. McCormick et al. confirmed the relation that smoking during pregnancy is linked to reduce birth weight [33]. Second hand smoke reduces weight gain and has a negative impact on the health of infants and older children. Nepal Demographic Health Survey, 2011 showed that 5 % of pregnant women and 7 % of breastfeeding women smoke cigarettes. Additionally, 4 % of pregnant women and 6 % of breastfeeding women consume other forms of tobacco.

Facility-based, population outreach, or home/family/community based antenatal, natal and postnatal care interventions have been proven to be effective to prevent infant deaths [34–36]. Therefore, the availability and use of public health care services, the utilization of antenatal, postnatal checkups, facility delivery, desired pregnancy, and availability of caesarian section facilities were also important proximate determinants of infant mortality though most of them were not statistically significant in this analysis.

The analysis also found that there was no significant difference between age of the mother and infant mortality though there is a high prevalence of early marriage and early pregnancy in Nepal. In line with this finding, an analysis of the World Fertility Survey data [37] showed that older maternal ages were not detrimental to infant survival. However, there was association between birth rank and birth interval. In fact, maternal fertility-related factors have an important influence on infant survival [26, 37, 38].

The identification of key determinants of infant deaths is important to provide guidance for the development of evidence-based focused interventions. In line with this need, National Health Policy, 2014 and Nepal Health Sector Program (2015–2020) have provisioned equity as a guiding principle of health programs. For this, as Buyana suggests, local government budgeting should be in a two-fold framework that combines both disease-based health needs and socio-economic needs [39]. Thus, it is important in Nepal to look upstream to address the causes in a holistic and integrated manner for social justice and universal coverage of health.

### Limitations

This paper included live-births, occurred within the 5 years preceding the survey. The associations of infant mortality with factors drawn from statistical analyses might lack a temporal relationship. This is due to the cross-sectional design used in Nepal Demographic Health Survey, 2011, thus limiting causal inference. For example, current poverty is a proxy for past poverty. Finally, the data for the Nepal Demographic Health Survey, 2011 was collected at the individual and household levels. For the present analysis, only crude community level indicators (such as region and urban–rural residence) were used.

## Conclusions

The analysis of NDHS data (2006 to 2010) in this paper demonstrated that socioeconomic determinants are associated with infant mortality in Nepal. Specifically, poorer and middle class people and people who reside in the Mountain ecological region and Far Western development region had high infant mortality. However, determinants like gender and urban/rural residence were found to be statistically insignificant.

These socioeconomic determinants operated through a common set of proximate determinants such as size of babies at birth, birth interval or spacing associated with high infant deaths. Therefore, infant mortality is typically multi-factorial in causality and the cumulative consequences of interactions of social, economic and biological determinants, among others. Hence, findings point to address both socioeconomic and proximate determinants side by side. For this, comprehensive, long-term, equity-based public health interventions and immediate infant care programs are recommended. Moreover, this study recommends an advanced analytical study to explore the independent roles of key determinants of infant mortality in Nepal.
